# Give It AGO: The Search for miRNA-Argonaute Sorting Signals in *Arabidopsis thaliana* Indicates a Relevance of Sequence Positions Other than the 5′-Position Alone

**DOI:** 10.3389/fpls.2012.00272

**Published:** 2012-12-07

**Authors:** Christoph J. Thieme, Christian Schudoma, Patrick May, Dirk Walther

**Affiliations:** ^1^Max Planck Institute for Molecular Plant PhysiologyPotsdam-Golm, Germany

**Keywords:** *Arabidopsis thaliana*, miRNA, Argonaute proteins, sorting, RNA-protein interaction, machine learning, random forests, mutual information

## Abstract

The specific recognition of miRNAs by Argonaute (AGO) proteins, the effector proteins of the RNA-induced silencing complex, constitutes the final step of the biogenesis of miRNAs and is crucial for their target interaction. In the genome of *Arabidopsis thaliana* (Ath), 10 different AGO proteins are encoded and the sorting decision, which miRNA associates with which AGO protein, was reported to depend exclusively on the identity of the 5′-sequence position of mature miRNAs. Hence, with only four different bases possible, a 5′-position-only sorting signal would not suffice to specifically target all 10 different AGOs individually or would suggest redundant AGO action. Alternatively, other and as of yet unidentified sorting signals may exist. We analyzed a dataset comprising 117 Ath-miRNAs with clear sorting preference to either AGO1, AGO2, or AGO5 as identified in co-immunoprecipitation experiments combined with sequencing. While mutual information analysis did not identify any other single position but the 5′-nucleotide to be informative for the sorting at sufficient statistical significance, significantly better than random classification results using Random Forests nonetheless suggest that additional positions and combinations thereof also carry information with regard to the AGO sorting. Positions 2, 6, 9, and 13 appear to be of particular importance. Furthermore, uracil bases at defined positions appear to be important for the sorting to AGO2 and AGO5, in particular. No predictive value was associated with miRNA length or base pair binding pattern in the miRNA:miRNA* duplex. From inspecting available AGO gene expression data in *Arabidopsis*, we conclude that the temporal and spatial expression profile may also contribute to the fine-tuning of miRNA sorting and function.

## Introduction

Non-coding, small RNA molecules have been revealed as essential for sequence-specific gene regulation in a broad spectrum of biological processes ranging from development, biotic, and abiotic stress response to modification of chromosomal structure (Reinhart et al., 2002; Carrington and Ambros, 2003; Bartel, 2004; Molnar et al., 2011). MiRNAs that act as post-transcriptional regulators of gene expression via degradation of specific target mRNAs or via the inhibition of their translation, constitute a well-studied class of small functional RNAs (Fire et al., 1998; Axtell et al., 2011; Mateos et al., 2011; Lee et al., 2012). Typically, miRNAs are between 20 and 24 nucleotides (nt) long and are known to interact with proteins of the Argonaute (AGO) family (Carrington and Ambros, 2003; Vaucheret et al., 2004; Joshua-Tor and Hannon, 2011). Both the miRNA and AGO protein constitute the essential part of the RNA Induced Silencing Complex (RISC), in which the miRNA guides the function of the AGO effector protein by providing the sequence complementarity-based recognition signal that allow the AGO to act on specific targets (Vaucheret, 2008; Joshua-Tor and Hannon, 2011).

Plant miRNAs are transcribed by RNA-polymerase II into primary transcripts called pri-miRNAs (Bartel, 2004; Mateos et al., 2011). In a first cleavage step, performed by DICER-LIKE1 (DCL1), characteristic hairpin-shaped precursors (pre-miRNAs) are produced. A second cleavage by DCL1 excises a duplex of typically 21-nt long mature miRNA (the guide strand) and the complementary bound miRNA star strand (miRNA*, the passenger strand) with both sequences each with a 2-nt-overhang at their 3′-end, respectively. This duplex is assumed to be exported from the nucleus into the cytoplasm, where the mature miRNA is loaded by an unknown mechanism into the RISC. Usually, after strand selection, the miRNA* strand becomes inactive and is degraded. However, recent studies demonstrated a biological function of the miRNA* strands (Devers et al., 2011; Yang et al., 2011; Zhang et al., 2011) and with release 17 of miRBase (Griffiths-Jones et al., 2008) the “mature/star” nomenclature was replaced by a “5p/3p” naming convention.

Argonaute proteins are considered to be the most important proteins of the mature RISC (Bohmert et al., 1998; Vaucheret, 2008). AGOs contain four domains: a variable N-terminal domain and the more strongly conserved PAZ, MID, and PIWI domains joined by the two linker domains L1 and L2. AGO proteins fold into a bilobal structure with a central groove for substrate binding, i.e., the small RNA molecule (Wang et al., 2009). The nucleotide specificity loop lining the binding pocket in the MID domain, recognizes the 5′-nucleotide of the small RNA (Frank et al., 2012) and the PAZ domain binds the 3′-terminal end of the RNA molecule (Wang et al., 2008). The PIWI domain adopts an RNaseH-like fold and can exert an endonuclease activity on the RNA target molecule identified by the bound small RNAs via sequence complementarity (Song et al., 2004; Rivas et al., 2005; Wei et al., 2012).

Argonautes participate in distinct RNA-interference (RNAi) pathways depending on the ribonuclease efficacy of the PIWI domain (Okamura et al., 2004; Qi et al., 2006). Of the potential ways in which small RNAs can act on their targets including silencing, RNA cleavage, translational repression, and transcriptional silencing, most miRNA-associated AGOs in plants were found to have the potential for target mRNA cleavage (Voinnet, 2009). However, cases in which translation is inhibited, similar to miRNA silencing in vertebrates, have also been reported (Brodersen et al., 2008). Several AGOs carry out multiple functions, e.g., AGO4 performs RNA-directed DNA methylation and also carries a slicer activity (Qi et al., 2006; Chellappan et al., 2010; Havecker et al., 2010).

The genome of the model plant *Arabidopsis thaliana* genome encodes ten AGO paralogs (named AGO1 to AGO10), assigned to three major evolutionary clades: the AGO1, AGO5, and AGO10 clade, the AGO2, AGO3, and AGO7 clade, and the AGO4, AGO6, AGO8, and AGO9 clade (Vaucheret, 2008; Joshua-Tor and Hannon, 2011). The grouping of AGOs to different clades is based on sequence distance measures and therefore AGOs belonging to the same clade may not necessarily share identical functions.

Argonaute1 was found to be the most essential AGO protein in the miRNA pathway (Vaucheret et al., 2004). AGO1 preferentially associates with 21–22-nt sequences with a 5′-uridine residue. Aside from binding miRNAs, AGO1 also associates with different classes of siRNAs and is involved in miRNA-induced ta-siRNA generation, a process termed transitivity (Manavella et al., 2012). AGO5 is assumed to carry out similar functions as its paralog, AGO1. By contrast, AGO10 (also referred to as ZWILLE or PINHEAD) specifically associates with members of the miR165 and miR166 families (Mallory et al., 2009; Zhu et al., 2011). This way, AGO10 is shown to withdraw those two miRNA families from the processing by AGO1 leading to their attenuation.

Even though AGO2 belongs to a different clade than AGO1, it also binds to miRNAs and siRNAs and it is suggested to perform functions that are largely redundant with AGO1 (Takeda et al., 2008; Maunoury and Vaucheret, 2011). In case of miR408, a double mutant of AGO1 and AGO2 is required for its suppression to avoid mutual compensation of both AGOs (Maunoury and Vaucheret, 2011). Compared to AGO1, AGO2 binds a high proportion of miRNA star strands (Zhang et al., 2011). Additionally, AGO2 is supposed to have an antiviral role as it associates with several virus-derived siRNAs (Takeda et al., 2008). AGO3 is closely related to AGO2 (Zhang et al., 2011). Both show high sequence similarity and adjacent localization in the genome and are proposed to share functions. AGO7, the third member of this clade, exclusively associates with miR390 and is required for TAS3 (trans-acting siRNA locus 3) dependent ta-siRNA production (Montgomery et al., 2008). AGO4 proteins regulate transcriptional gene silencing (TGS) by RNA-directed DNA methylation and are primarily associated with 24-nt siRNAs (Qi et al., 2006; Havecker et al., 2010). Additionally, AGO4 is also involved in RNA cleavage and is shown to trigger ta-siRNA generation, e.g., by miR172 and miR390 (Qi et al., 2006; Montgomery et al., 2008). Like AGO4, the other members of the clade, AGO6 and AGO9, specifically act in DNA methylation pathways and TGS (Zheng et al., 2007). AGO8 shows low-level expression in all stages and tissues and thus is considered to be a pseudogene (Takeda et al., 2008; Mallory and Vaucheret, 2010).

Thus, different AGO proteins are associated with different functions, and even in cases of redundant function, their efficacy differs (Okamura et al., 2004; Capitao et al., 2011; Joshua-Tor and Hannon, 2011). Hence, a precise sorting of small RNAs into the appropriate AGO complex, a process referred to as AGO sorting, is essential for their biological function.

Experimental studies have shown that different AGOs indeed preferentially bind specific miRNAs (Mi et al., 2008; Montgomery et al., 2008). The signal for this sorting is presumed to reside in specific nucleotide sequence and structural features of the small RNAs (Kim, 2008; Mi et al., 2008; Czech and Hannon, 2011). The 5′-terminal nucleotide has been identified to act as the crucial signal with regard to AGO sorting (Kim, 2008; Mi et al., 2008; Takeda et al., 2008). Most miRNAs are incorporated into an AGO1-based RISC and start with the corresponding 5′-terminal uridine (Takeda et al., 2008). By contrast, siRNAs typically carry adenosine residues at their 5′end and are preferentially incorporated into AGO4. The central role of the 5′-nucleotide has also been corroborated by additional experiments that showed that AGO1-associated small RNAs are enriched for molecules that contain a 5′-uridine, whereas AGO2, AGO4, AGO6, and AGO9 primarily bind to small RNAs starting with an adenosine residue (Mi et al., 2008; Zhu et al., 2011). While AGO5 preferentially incorporates small RNA sequences showing 5′-terminal cytidines, binding analyses to nucleotide monophosphates have revealed that this association is less strict and 5′-adenosine as well as 5′-guanosine are bound with similar affinities (Frank et al., 2012). For AGO7, mainly associated with miR390, no preference for a particular 5′-terminal nucleotide could be identified (Montgomery et al., 2008). AGO9 was suggested to be primarily associated with 5′-adenosine small RNAs (Havecker et al., 2010). AGO10 predominantly associates with members of miR165/166 family containing a 5′-uridine (Zhu et al., 2011).

However, in view of the different functions associated with different AGOs, a sorting system based solely on the nature of the 5′-nucleotide (i.e., on an alphabet of only four letters allowing to encode four different signals only) appears not specific enough and underdetermined. Thus, sequence or structural features beyond the 5′-terminal residue appear necessary to ensure unambiguous miRNA sorting. In addition, several substantial exceptions from the 5′-terminal rule have been reported. While mutation experiments of the 5′-nucleotide confirmed the importance of the first position by redirecting miRNAs from AGO1 toward AGO2 by exchanging the 5′-nucleotide and the reverse, the same experiments, also revealed several cases, where the assignment to an AGO appeared to be based on different attributes such as base pair mismatches or interactions with other proteins (Mi et al., 2008; Montgomery et al., 2008). The members of the miR165/166 families contain a 5′-uridine, but are specifically associated with AGO10 instead of AGO1 (Zhu et al., 2011). MiR390 contains a 5′-adenosine and is selectively chosen by AGO7 (Montgomery et al., 2008), whereas miR408, also starting with an adenosine, promiscuously associates with AGO1 and AGO2 (Maunoury and Vaucheret, 2011). AGO4, AGO6, and AGO9 associate primarily with 5′-adenosine siRNAs and the mechanism of their AGO sorting remains unclear (Havecker et al., 2010). The presence of multiple different AGOs in other plant genomes further supports the notion of the existence of a more versatile sorting code than relying on a single sequence position alone. For example, the genome of *Oryza sativa* encodes 19 AGO paralogs (Kapoor et al., 2008), 10 are known in *Populus trichocarpa*, and 6 in *Physcomitrella patens* (Wei et al., 2012).

In this study, we set out to revisit the issue of AGO sorting. We analyzed a high-quality dataset of miRNA-AGO sorting events based on published high-throughput sequencing of RNAs combined with crosslinking-immunoprecipitation (HITS-CLIP) data in *A. thaliana* (Mi et al., 2008). First, we investigated whether AGO sorting has a functional relevance for miRNA action also from the perspective of the putative targets. By applying various correlation approaches such as mutual information (MI) and methods from machine learning, we aimed to identify additional sequence-related features that may determine the AGO sorting in *Arabidopsis*. Furthermore, we probed the relevance of the secondary structure of the miRNA:miRNA* duplex and its influence on the affinity to an AGO protein. Additional factors that are not related to the mature miRNA itself, such as sequence motifs up or downstream of the mature miRNA along the miRNA precursor sequence to which co-factors may bind, may also play a critical role for the specific AGO-miRNA recruitment. We applied motif recognition approaches to identify such motifs. Because the sorting process may simply be regulated by the differential expression of the respective AGO gene, the influence of spatial and temporal expression of miRNAs or the corresponding AGO has been taken into consideration as well.

Our results suggest that in addition to the 5′-position, other sequence position across the entire length of miRNA sequences are informative for the sorting process as well.

## Materials and Methods

### Sequence data, mapping, and candidate miRNA selection

We retrieved *A. thaliana* mature and precursor miRNA sequences from miRBase (release 18, November 2011; Griffiths-Jones et al., 2008). We applied RNAhybrid (Kruger and Rehmsmeier, 2006) to find the sections of miRNA and miRNA* on the precursor and to infer the pattern of paired and unpaired bases from the minimum free energy (MFE) structure. For our analyses, we used the complementary sequences of the miRNA:miRNA* duplex, ignoring the 3′-overhangs.

In accordance with Nozawa (Nozawa et al., 2012) and the miRBase annotation guidelines (Meyers et al., 2008), we excluded spurious miRNAs. Specifically, we discarded miRNAs if their precursor contained more than six mismatches or a bulge of more than three nucleotides within the predicted miR/miR* section.

### Experimentally identified AGO sorting of Ath-miRNAs

We obtained a set of experimentally identified *Arabidopsis* miRNA-AGO pairs from published high-throughput sequencing data of RNA isolated in crosslinking-immunoprecipitation (co-IP) experiments (Mi et al., 2008; GEO accessions GSM253622, GSM253623, GSM253624, GSM253625). The dataset included RNA sequence reads associated with AGO1, AGO2, AGO4, and AGO5. Adapter sequences were removed and all reads trimmed to 30-nt length by using Trimmomatic (Lohse et al., 2012). We applied Bowtie (Langmead et al., 2009) for exact mapping of all known Ath-miRNAs contained in miRBase to the sequencing reads (end-to-end mapping using seed length of 5) resulting in 3,241,388 total read counts for AGO1, 771,808 for AGO2, 2,148,570 for AGO4, and 874,751 for AGO5. Read counts associated with particular miRNAs as determined by mapping were normalized to the total number of reads per AGO multiplied by 1 million (RPM). MiRNA sequences covered by less than 10 RPM-reads were excluded from further analysis. We considered miRNAs with more than 70% of their associated reads in a particular AGO co-IP fraction to be preferentially bound by the respective AGO complex.

Of the 328 *A. thaliana* miRNAs contained in miRBase and after filtering, 148 were also contained in the published co-IP data set. According to our criteria, 70 unique miRNA sequences were found to be preferentially sorted to AGO1, 25 miRNAs to AGO2, and 22 miRNAs to AGO5. Only nine miRNA sequences could be identified to be specifically processed by AGO4. Furthermore, 22 miRNAs did not display any preference for any AGO class with associated sequencing reads being found to co-precipitate with several AGO proteins. Due to the low number of observations, AGO4-specific miRNAs were omitted from many statistical analyses of the AGO sorting process presented in this study. Hereafter, we refer to the set of non-redundant miRNAs with a clear and experimentally identified preference toward a single AGO as the *Confidence Set*. Unless otherwise stated, all miRNA sequences were trimmed to length 21 nucleotides rendering them length-identical.

### miRNA-target prediction and gene ontology term enrichment analysis

For the miRNA sequences, we predicted potential targets using psRNA Target (Dai and Zhao, 2011) on the TAIR10 (Lamesch et al., 2012) cDNA dataset applying default parameters. TAIR locus IDs (accession numbers) were extracted for all targets and grouped according to the AGO mapping of the corresponding miRNA. We compared each set of targets for miRNAs preferentially bound by AGO1, AGO2, or AGO5, to the target set associated with the respective other AGOs. We obtained plant Gene Ontology (GO) slim terms for function, process, and component from TAIR and GO-term enrichment analysis was performed using Fisher’s exact test with subsequent False Discovery Rate (FDR) multiple testing correction to the obtained *p*-values according to (Benjamini and Hochberg, 1995). We required the *p*-values of the results to be lower than 5% for reporting. To minimize the bias from large miRNA families with similar sequences and therefore similar targets on the GO profiling results, we truncated miRNA sequences from the *Confidence Set* to 20-nt and discarded duplicate 20-mers from the analysis prior to target mapping, thus, ensuring a sufficient density of mismatches.

### Mutual information computations

The MI between all miRNA sequence positions and the AGO class vector was computed as:

$$\begin{array}{llll} & MI\left(AGO_{V};SeqV_{i}\right) & & \\ & =\sum_{ago\in AGO_{V},base\in SeqV_{i}}P\left(ago,\;base\right)*\log_{2}\frac{P\left(ago,\;base\right)}{P\left(ago\right)*P\left(base\right)}, & & \text{(1)} \end{array}$$

where *ago* denotes a particular AGO class (1, 2, or 5), *base* one of the four possible nucleotides (A, C, G, or U), *AGO_V_* the vector of all AGO assignments, and *SeqV_i_* the sequence vector taken as the *i*-th column (sequence position) from the 5′- and non-gapped aligned miRNA sequences from the *Confidence Set* paired up with their respective AGO. *P* denotes the probability of joint (*ago* and *base*) or individual occurrences (*ago* or *base*). We obtained empirical *p*-values by comparing the actual MI value to the distribution of MI values obtained from 10,000 repeat runs taking label shuffled vectors; i.e., the AGO assignments were randomized and the MI values computed anew. As 21 positions in the alignment of miRNA sequences were tested, we adjusted the individual, position-specific *p*-values by FDR multiple testing according to Benjamini and Hochberg (1995).

Inspecting the available three-dimensional structural information of AGO proteins revealed that 5′-ends of small RNAs are anchored in a loop region of the MID domain of AGO proteins, where hydrogen-bonds of the peptide side chains have been shown to mediate 5′-nucleotide specificity (Frank et al., 2012). As most interactions between the protein and the small RNA take place in this binding pocket (Wang et al., 2009; Frank et al., 2012), we assumed all mature miRNA sequences to be anchored in this pocket and thus treated them left-aligned on their 5′-terminal nucleotide.

### Prediction of AGO sorting using random forests

We applied the Random Forest (RF; Breiman, 2001) classification method as implemented in the R package randomForest (Liaw and Wiener, 2002) to assess non-linear, multivariate dependencies of different miRNA features. To account for unequal set sizes (Table 1), we used sample sizes of 20 (parameter *sampsize*) to grow each tree for the three-class (AGO1, 2, and 5) prediction problems. Default parameters for the number of variables employed in splitting each node (*mtry*) were used. The default number of trees to be grown was used. We trained RF models on two different input sets of features based on the sequence and secondary structures of mature miRNAs from the *Confidence Set*. We used (1) the 5′-aligned 24-nt miRNA sequence (with shorter sequences 3′-padded with “N”), and (2) the pattern of bound (i.e., canonical Watson–Crick base-pairing), unbound, and wobble pairings in the miRNA:miRNA* duplex (for sequences shorter than 24-nt, the 3′-end was assumed to be unbound). To eliminate the already known impact of the first nucleotide position on the AGO sorting and to specifically identify additional classification signals along the remaining miRNA sequence positions, the first position was left out in specified cases.

**Table 1:** **Base composition of the 5′-positions of all *Arabidopsis thaliana* (Ath) miRNAs as contained in miRBase and for sequence-unique miRNA found to be specifically associated with AGO proteins 1, 2, 4, and 5, respectively**.

	A	C	G	U	Total unique miRNAs
Ath miRNA	66	27	21	214	328
**AGO**					
1	0 (0.0)	6 (1.04)	1 (0.2)	63 (1.4)	70
2	21 (4.2)	0 (0.0)	1 (0.6)	3 (0.2)	25
4	5 (2.7)	0 (0.0)	0 (0.0)	4 (0.7)	9
5	0 (0.0)	6 (3.3)	0 (0.0)	16 (1.1)	22

*Listed are the absolute counts as well as the corresponding odds ratios. Odd ratios were obtained by dividing the relative frequency of a particular 5′-base for a given AGO class by the corresponding relative 5′-base frequency in all Ath miRNAs. Odds ratios less than 0.5 (i.e., less than half than expected based on the general counts) are highlighted blue, while odds ratios greater than 2 indicating enrichment of a particular base relative to the background distribution are colored red. Note that the counts refer to unique sequences and not read counts as provided by Mi et al. (2008) to eliminate the influence of expression level*.

We computed the accuracy of RF classifications defined as the quotient of correct class assignments and the total number of assignments obtained from the “out-of-bag” (OOB) predictions; i.e., the standard internal RF cross-validation based on bootstrapping was used. The margins associated with each prediction served as prediction scores. The margin is defined as the proportion of votes for the correct class minus the maximum proportion of votes cast for an alternative class. For assessing the predictive power associated with the actual miRNA sequences, we generated randomized datasets based on class shuffling.

Statistical significance of differences of the prediction accuracy associated with different sets (actual vs. randomized or for comparing different feature input sets) was assessed by the non-parametric two-sample Wilcoxon rank-sum test on the margins of the respective data sets to be compared. The reported *p*-values were computed as the median *p*-value obtained in 1,000 repeated RF runs. As every run differs in the feature splits, the ensemble of trees, and the bootstrap samples (OOB), but uses the same original dataset size, the reported *p*-value can be regarded as a bootstrap estimate of the true *p*-value. While there remains a risk of amplifying peculiarities of the dataset, the reported *p*-value reflects the original dataset size and is not artificially decreased by computing the *p*-value only after all repeat runs.

The importance of the different features was assessed by the mean decrease variable importance metric, which captures the loss of predictive power by selectively permuting the values of each feature (here sequence position) individually.

### Motif detection

Base preferences at particular sequence positions in miRNAs sorted to AGOs 1, 2, and 5, respectively, were identified and visualized using sequence logos. Sequence logos were produced by the WebLogo 3 software available at http://weblogo.threeplusone.com (Crooks et al., 2004) using default settings for mature sequences from the *Confidence Set* trimmed to 21-nt.

Scans for over-represented motifs in the sequence regions upstream of the mature miRNA observed to be associated with a particular AGO were performed using MEME (Bailey et al., 2009) and Amadeus V1.2 (Linhart et al., 2008). We extended the miRNA precursor sequences by adding 500-nt from their genomic context in both 5′- and 3′-direction. Sequences generated in a similar fashion for miRNAs over-represented in the pools of the respective other two remaining AGO classes served as background in the motif scans. For MEME, zero or one motifs per sequence were allowed. Using Amadeus, we performed a search according to the “UTR scan for motifs in arbitrary organisms” protocol. For both tools, we allowed the length of potential motifs to be 6-nt.

### AGO expression in *A. thaliana*

Affymetrix microarrays (ATH1 22k GeneChip) were analyzed for spatial (anatomy-based) and temporal (development-based) differential expression of *A. thaliana* AGOs (AGO1: AT1G48410, AGO2: AT1G31280; AGO3: AT1G31290, AGO4: AT2G27040, AGO5: AT2G27880, AGO7: AT1G69440, AGO9: AT5G21150, AGO10: AT5G43810) using Genevestigator (Hruz et al., 2008). The applied hierarchical clustering for AGO expression was based on Pearson correlation applied to the normalized gene expression data as processed in Genevestigator.

## Results

As reviewed in the Introduction, the biological relevance of the sorting of miRNAs to specific AGO proteins has been discussed in the context of AGO-specific modes of target inhibition such as the siRNA or miRNA mechanisms. The need for a specific AGO sorting and, thus, the requirement for the existence of sorting signals associated with miRNAs or their precursor molecules has been derived from those observed differences in biological mechanisms and actions associated with individual AGO proteins. To further motivate the study of AGO sorting signals, we first performed a comparison of the miRNA targets associated with miRNAs that are processed specifically by particular AGO proteins in order to elucidate whether AGO-specific processing is associated with distinct target classes from a functional and subcellular localization perspective.

### AGO-specific biological action of miRNAs as judged by gene ontology enrichment analysis

From the published co-immunoprecipitation dataset (Mi et al., 2008), we extracted 70 miRNAs with a clear sorting preference for AGO1, 25 miRNAs for AGO2, and 22 miRNAs for AGO5 (see Materials and Methods). For the miRNAs of this *Confidence Set*, 416 potential targets were predicted for AGO1-associated miRNAs, 134 for AGO2-associated miRNAs, and 168 for AGO5-associated miRNAs. Even though the sets of miRNAs are mutually exclusive, several common targets were predicted nonetheless. AGO1- and AGO5-associated miRNA targets were found to share 26 targets. MiRNAs bound by AGO1 and AGO2 have only one target in common, and AGO2 and AGO5 share two targets. Assuming 30,000 *A. thaliana* genes, 2.3 targets are to be expected to be shared between AGO1 and AGO5 as a result of a purely random selection of genes, likewise 1.8 targets are expected to be in common between AGO1 and AGO2, 0.75 and for AGO2 and AGO5. Thus, the target sets of AGO1 and AGO5-miRNAs overlap to a significantly larger than expected degree, while the other AGO pairs are in line with random expectations. Thus, as judged by target overlap, no evidence was found for a distinct biological action of miRNAs processed by different AGO proteins. On the contrary, AGO1 and AGO5 appear to share more targets than randomly expected.

Next, we profiled the disjoint target sets; i.e., removing shared targets, to discern whether the respective AGO target groups can be distinguished by their biological process, function, or subcellular localization as captured by the available GO annotations for the target genes. Indeed, AGO1 targets appear to be enriched in targets associated with *developmental processes* (*p*_FDR_ = 1.9E-05) and to be involved in *transcription factor activity* (*p*_FDR_ = 4.66E-10). Furthermore, AGO1 targets are enriched in *nucleus* localizations (*p*_FDR_ = 1.4E-4). (Italicized words refer to the respective GO-slim terms). For AGO2 and AGO5, no enrichment of any GO-slim term was evident suggesting that the particular processes, functions, and locations associated with AGO2 and AGO5 targets are distributed relatively evenly among all three AGO target sets. Thus, from the target perspective, specific biological action necessitating a fine-tuned and precise sorting of miRNA to their AGO proteins could only be established for AGO1.

### Properties of AGO-specific miRNA sequences

**Figure 1:**
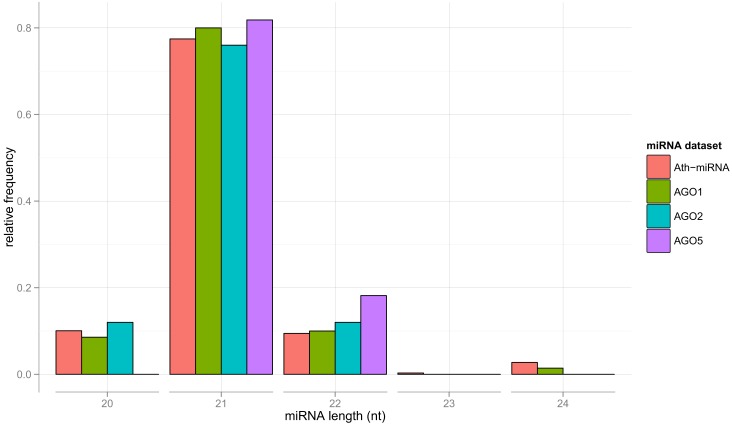
**Length distribution of all *Arabidopsis* (Ath) miRNAs as contained in miRBase, and for sequence-unique miRNA found to be specifically associated with AGO proteins 1, 2, and 5, respectively (the *Confidence Set*)**.

We now turn to the characterization of the miRNA sequences associated with particular AGO proteins in search for possible sorting signals. For the miRNAs contained in the *Confidence Set*, the length distribution closely resembles each other and is similar to the general length distribution of *A. thaliana* miRNAs contained in miRBase (Figure 1).

The base type that is observed most frequently at the 5′-position for all *A. thaliana* mature miRNAs currently listed in miRBase is uracil (214 occurrences), followed by adenine (66), cytosine (27), and guanine (21) (Table 1). As reported previously (Mi et al., 2008; Takeda et al., 2008), AGO1 shows a bias toward miRNAs with a 5′-uracil (Table 1). However, when compared to the background distribution of all miRNAs, the relative enrichment is 1.4-fold only (odds ratios in Table 1). By contrast, the 5′-position AGO2 processed targets exhibits a very strong enrichment of adenine nucleotides (4.2-fold) as does AGO4, albeit the statistical significance is lower given the small absolute count. Similarly, AGO5-miRNAs appear to be enriched in 5′-cytosines, but to also accept uridines (Table 1). Thus, based on the 5′-position alone, AGO1 appears to be compatible with the dominating 5′-uracil of miRNAs in general, whereas the 5′-terminal adenine may act as a sorting signal for AGO2 and AGO4, and likewise, cytosine for AGO5 as reported previously (Kim, 2008; Mi et al., 2008). However, a substantial ambiguity remains as a large number of miRNAs are processed by AGO proteins with 5′-terminal bases deviating from this simple scheme (Table 1). Furthermore, relying on a single position only would only allow for four different AGOs to be targeted specifically given the four possible different nucleotide bases. Thus, the presence of additional sorting signals that may further specify the 5′-position code appears necessary.

### Search for informative miRNA sequence positions by mutual information

**Figure 2:**
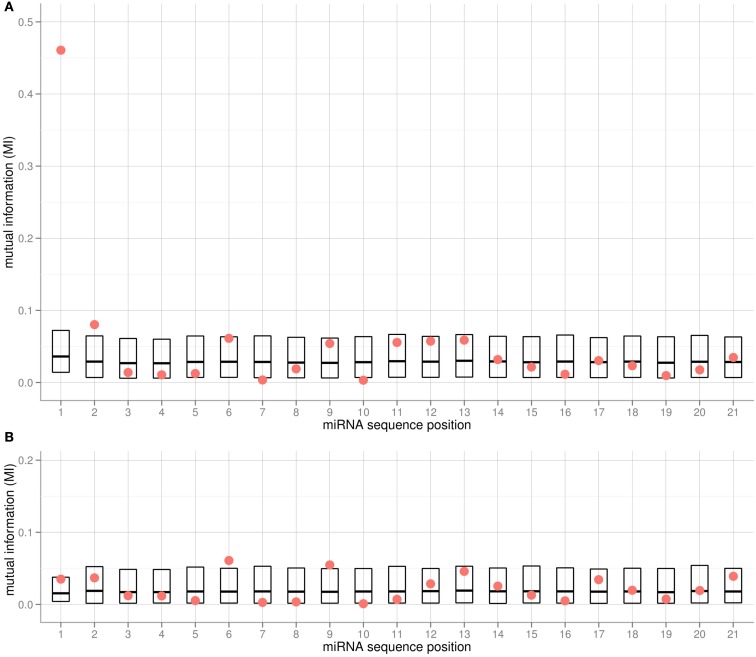
**Mutual Information (MI) for each alignment position of mature miRNA sequences from the *Confidence Set* and the associated (A) AGO1, AGO2, and AGO5, and (B) AGO1 and AGO5 only**. Red dots represent the actual MI values. For estimating statistical significance, the MI values of shuffled data are also provided (boxes showing 5% percentile, mean and 95% percentile of 10,000 iterations). In **(A)** for position 1, the actual MI is significantly higher than for the MI shuffled data (*p*_FDR_ < 0.0021). Note that the shuffling was done per position, such that different base compositions leading to different background distributions are taken into account.

We applied the MI metric as an effective means to assess the co-segregation of nucleotides and associated AGO proteins – both categorical variables – along all positions of the 5′-aligned miRNA sequences. Any significant correlation between the type of nucleotide at a given position and the chosen AGO would be signified by high MI values. To gauge significance, all MI values were compared to random MI values obtained from shuffling experiments. Evidently, the 5′-position of miRNAs is most informative with regard to the chosen AGO (Figure 2A). In addition, positions 2, 6, 9, and 11–13 were found to be associated with relatively high MI values as well. However, none of the respective MI values remained statistically significant after accounting for multiple testing (21 tests according to the number of positions in the miRNA alignment). Computing the MI values considering only two instead of all three AGO proteins (e.g., considering AGO1 and AGO2 and associated miRNAs only) resulted in similar MI profiles, with the exception of the pair AGO1 and AGO5. Here, the MI associated with the 5′-position is not significant (Figure 2B, *p* = 0.11, *p*_FDR_ = 0.4) as both AGOs accept uracil bases in this position (Table 1). Interestingly, positions 6 and 9 were found with increased MI values (Figure 2B, *p* = 0.026, *p*__FDR_ = 0.34 and *p* = 0.034, *p*__FDR_ = 0.36, respectively), thus possibly serving as additional sorting signals to help resolve the ambiguity associated with the 5′-position for the AGO1 vs. AGO5 sorting decision. In conclusion, while a few sequence positions along the miRNA appear to carry some information with regard to the AGO sorting, convincing statistical significance could only be established for sequence position 1; i.e., the 5′-terminal position.

The miRNA molecules may be bound by the AGO proteins as a miRNA:miRNA* duplex (see Discussion on this point). As there is no perfect one-to-one correspondence of the mature sequence and the star-sequence because of mismatches and deviations of canonical base-pairing (see below, Figure 4), the star-sequence may carry different information, and may, in fact, contribute the sorting signal. However, applying the MI-analysis to the star strand sequence associated with every miRNA did not yield any significant MI-peaks. On the contrary, the MI value found for the miRNA* position that is opposite to the first position of the mature strand is much less informative (*p*_FDR_ = 0.47).

### AGO sorting sequence signatures

**Figure 3:**
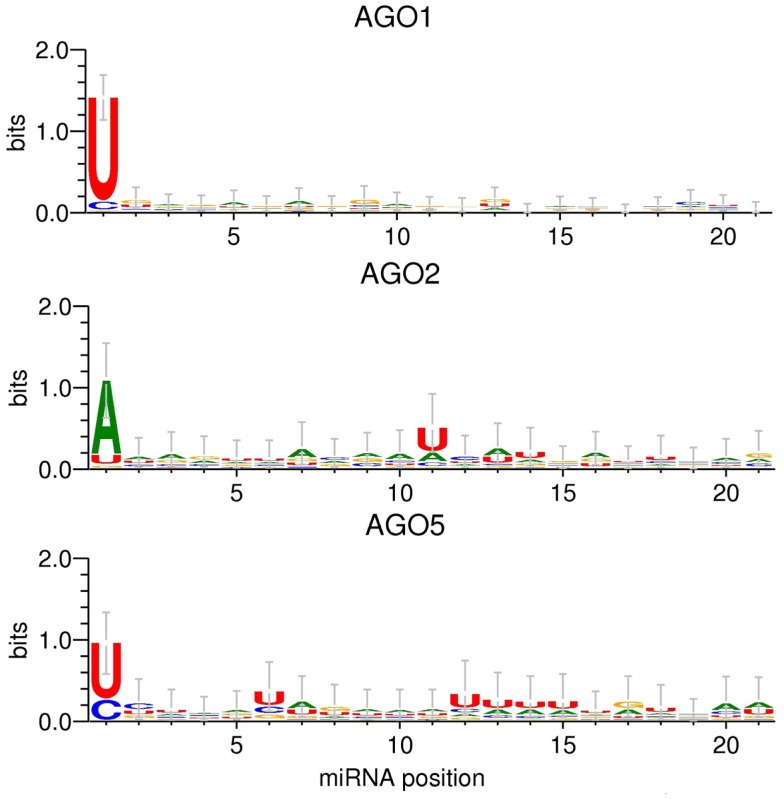
**Sequence logo presentations of motifs for miRNAs of the *Confidence Set* that are preferentially associated with AGO1, AGO2, and AGO5**. The height of the stack of symbols or individual base signifies the level of conservation of the given position or base type, respectively, expressed as information contents. Information contents of two bits would correspond to a position exclusively occupied by a single base type. Error bars correspond to Bayesian 95% confidence intervals as estimated by the WebLogo 3 tool (Crooks et al., 2004).

The applied MI approach gauges the significance of single positions relative to the AGO sorting position, one at a time. Even though this analysis did not yield any statistical evidence for the relevance of any other but the first sequence position for the AGO sorting decision, visualizing the actual base compositions along the miRNA sequence positions may still provide an impression as to whether a combination (additive or conditional) of several sites may turn out to be informative. [We will report on the more rigorous search for such higher-order sorting signals below (RF classification)]. Figure 3 shows the sequence logos obtained for the sequence sets associated with AGO 1, 2, and 5, respectively. In essence, sequence logos visualize the base frequencies (their “conservation”) at different positions along with their information content.

The 5′-sequence position (position 1) shows the most pronounced AGO-specific base preferences (Table 1). In addition to the characteristic 5′-uridine, AGO1-miRNAs display no apparent compositional preferences at any other sequence position. Aside from the typical adenine at the 5′-sequence position, AGO2-miRNAs exhibit an increased frequency of uridine at position 11. In the AGO5 dataset, uridine residues are found at increased frequencies at position 6 and 12–15, and most pronounced at the 5′-position (Figure 3). The comparison of the sequence logos associated with AGO1 vs. AGO5-miRNAs appears to suggest that, while no single position proved statistically informative using the MI analysis and as also apparent from the error bars in Figure 3, an enrichment of particular base types (uridines) associated with AGO5 at several positions may – in combination – still yield enough information to serve as a sorting signal. Thus, even though both miRNA sets are characterized by the same 5′-uridine potentially causing ambiguous sorting, AGO5 sequences may still be distinguishable based on the combination of several other sites. We will turn to the identification of such higher-order motifs below by applying the RF classification approach.

### Base-pairing patterns as a potential sorting signal

**Figure 4:**
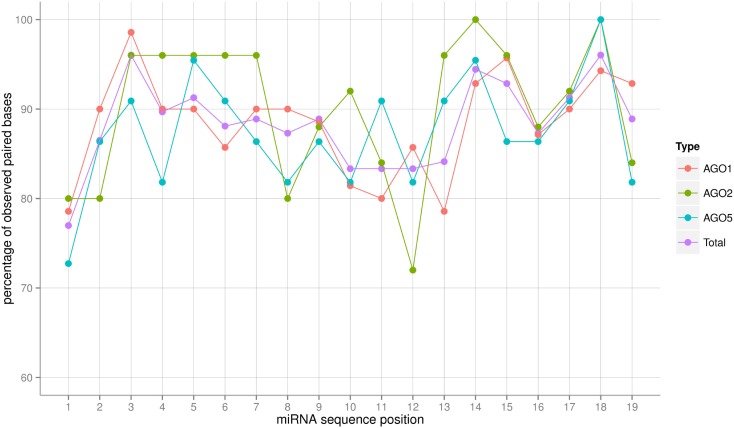
Frequency in percent of base-pairing (Watson–Crick or G:U wobble pairs) for each position of the miRNA:miRNA* duplex as predicted by RNAhybrid. The frequency was obtained by dividing the number of sequences in which base-pairing occurred at a given position by the total number of miRNA sequences associated with a particular AGO. “Total” refers to the average of a combined set of all AGO1, AGO2, and AGO5 sequences. Connecting lines are included for visualization purposes only. Note that because the overhanging 3′-end of the mature miRNA sequence, sequence positions up to position 19 were considered only. For those positions, miRNA:miRNA* duplex formation can be assumed as all miRNAs in the set were of length 21 or greater.

The AGO sorting process constitutes a specific recognition event between a protein (the AGO) and a (likely) double-stranded RNA (the miRNA:miRNA* duplex) molecule (see Discussion on this point). Assuming an RNA-duplex with helical structure via base-pairing, different miRNA sequences would result in almost no changes of the interaction surface as the helical shape is maintained and only subtle electrostatic differences (hydrogen-bond forming potential) would have to be responsible for specific AGO protein binding. However, larger structural alterations brought about by deviations from canonical base-pairing could potentially lead to more substantially changed interaction surfaces and thus may serve as a sorting signal. We inspected the degree of sequence complementarity allowing canonical Watson–Crick or wobble base-pairing across the full-length of the miRNA sequence (Figure 4). The miRNAs sorted to the three different AGO proteins considered in this study appear to follow the same base-pairing pattern along their sequence with differences most likely caused by the fluctuations associated with low numbers of observations. Perfect base-pairing seems to be required at positions 3, 14, 15, and 18, whereas unpaired nucleotides seem to be tolerated at positions 1 and 10–13. If at all, then the AGO2-miRNAs appear to possess the most characteristic base-pairing profile compared to AGO1 and AGO5. AGO2-miRNAs seem to require a perfect stem section; i.e., perfect base-pairing, at positions 3–7. By contrast, in seven of the 25 AGO2 co-IP miRNAs, position 12 of the duplex is not involved in a Watson–Crick or G:U wobble base pair. Nonetheless, we conclude that base-pairing differences leading to altered interaction surfaces are likely not providing a sorting signal. Interestingly, position 1 exhibits a low base-pairing tendency in all three AGO-miRNAs types, which is consistent to the notion that the 5′-position is specifically recognized by AGO proteins and thus needs to be structurally more accessible. This is achieved by a decreased involvement in base-pairing (Wang et al., 2008; Frank et al., 2012).

### Higher-order sorting patterns – random forest classification

So far, we have focused on determining the relevance of *individual* miRNA positions and the correlation with selected AGO. Consequently, the search has concentrated on univariate properties, one position at a time. However, any interactions between positions have not yet been considered. For example, it is conceivable that AGO-specific recognition requires two or more positions to be occupied by a specific combination of bases. To reveal such possible higher-order patterns and their effect on AGO sorting, we applied RF, a tree-based classification method to the prediction of AGO proteins based on miRNA features. With regard to considered features, we used (i) the sequence information for 5′-aligned miRNA sequences; i.e., the occupancy of particular positions by a given base, and (ii) base pair binding patterns as discussed above.

As reported in the literature (Kim, 2008; Mi et al., 2008; Montgomery et al., 2008) and as is also evident from the base composition statistic (Table 1), the 5′-position is indeed predictive of sorting, albeit at 52.4% accurate predictions only, (Case C, Table 2) caused by the many ambiguities associated with relying on the 5′-position alone (e.g., AGO1 vs. AGO5 both accept uridines). However, by adding the information associated with all remaining miRNA sequence positions, the prediction accuracy was boosted significantly to 63.6% (Case D, Table 2). Likewise, predictions based on all sequence positions but the first position (Case B, Table 2) also yielded significantly better (42.1% accuracy) than random predictions (as expected, 33% accuracy for the three-class prediction problem, Case A, Table 2). Thus, using the RF classification approach, the predictive value of the whole miRNA sequence, and not only the first position alone, was unveiled. By contrast, relying on base pair binding patterns; i.e., utilizing the secondary structure information for the miRNA:miRNA* duplex, no significant performance gain relative to random predictions was obtained (Case E, Table 2).

**Table 2:** **Accuracy of Random Forest predictions for the sorting of miRNAs from the *Confidence Set* to either AGO1, 2, or 5**.

	(A) Class- shuffled	(B) All sequence positions except first	(C) First position only	(D) All sequence positions (including first position)	(E) Binding pattern (all positions including first)
RF prediction accuracy (%)	33	42.1	52.4	63.6	38.7
*p*-value (compared sets)		0.049 (B vs. A)		0.022 (D vs. C)	0.132 (E vs. E-shuffled)

*Case (A), random prediction accuracy for class-label shuffled data and sequence input excluding position 1, i.e., taking for sequence positions 2 and onward only. Case (B), predictions for sequence positions 2 and onward; i.e., omitting the first, 5′-position. Case (C), predictions taking the actual base in the first position augmented by 23 random base calls. All miRNA sequences were taken as length 24, and if shorter padded by “N”-characters*.

**Figure 5:**
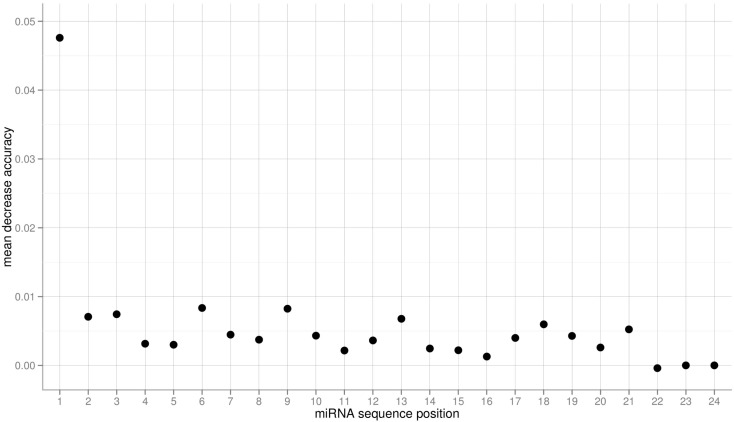
**Variable importance measure computed as the mean decrease of accuracy for the three-class RF prediction of AGO sorting using the sequence information of miRNAs of the *Confidence Set***. Here, larger values indicate increased importance for the classification decision.

The obtained variable importance metric associated with all sequence positions (Figure 5), identified position 1 to carry the most information by far. Consistent with elevated MI values found at those positions, secondary peaks are found at position 2, 6, 9, 18, and 21 (Figure 5). No importance was found for sequence positions 22 or greater. As those positions essentially capture miRNA sequence length (miRNAs shorter than 24-nts were padded with “N”s), we conclude that miRNA sequence length is not predictive of the AGO sorting as evident already from the nearly identical length distributions of miRNAs with sorting preferences for different AGOs (Figure 1).

### Informative positions in the context of the 3D-structure of AGO proteins

The available crystal structure of the full-length AGO protein of *Thermus thermophilus* allows correlating the MI profile with specific structural contacts along the miRNA sequence (Wang et al., 2008). (Note that for *Arabidopsis*, only the structure of the MID domain has been determined such that large interaction surface regions are missing. Furthermore, no structural information was included for the miRNA molecule, but for single nucleosides only; Frank et al., 2012). Based on the published hydrogen-bonding pattern between the miRNAs (positions 1–15) and AGO protein amino acid residues (Suppl. material of Wang et al., 2008), we correlated the position-specific MI values to the number of hydrogen-bonds reported for the equivalent position in *T. thermophilus* and obtained *r*_Pearson_ = 0.7135 (*p* < = 0.01), and *r*_Spearman_ = 0.2216 (*p* = 0.21), respectively. The relatively large difference between the Pearson and Spearman-correlation coefficients can be attributed to the 5′-position that exhibits both high MI score and high number of hydrogen-bonds and thus acts as an outlier. Nonetheless, embedding the MI profile into the structural context supports the notion that the hydrogen-bond network may guide the AGO selectivity and the dominating role of the 5′-miRNA-position.

### Search sequence motifs outside the mature miRNA sequence

It appears possible that the AGO sorting is influenced by protein co-factors that bind to sequence motifs on the miRNA precursor sequence outside the mature miRNA sequence and subsequently guide the miRNA to its specific AGO. Therefore, as another option for a potential sequence-based sorting signal, we searched for over-represented short sequence motifs in up and downstream genomic regions relative to the position of the mature miRNA in comparison to equivalent sequence sets for miRNAs preferentially consumed by the respective other remaining AGOs. However, despite using relaxed thresholds, neither searching by MEME nor Amadeus yielded any significant AGO-specific motif in the sequence context of mature miRNAs, neither up to 500-nt up or downstream nor within the precursor itself.

### Potential of AGO sorting via differential expression of the AGO genes

As an alternative to sorting signals associated with the miRNAs and their sequences themselves, differential expression of AGO genes may result in the observed sorting preferences. Sorting could be accomplished by differentially regulating AGO and miRNA gene expression, and subsequently, the particular AGO protein that is expressed would bind rather unspecifically to any miRNA currently present in the cell effectively resulting in a sorting of miRNA to AGO proteins.

**Figure 6:**
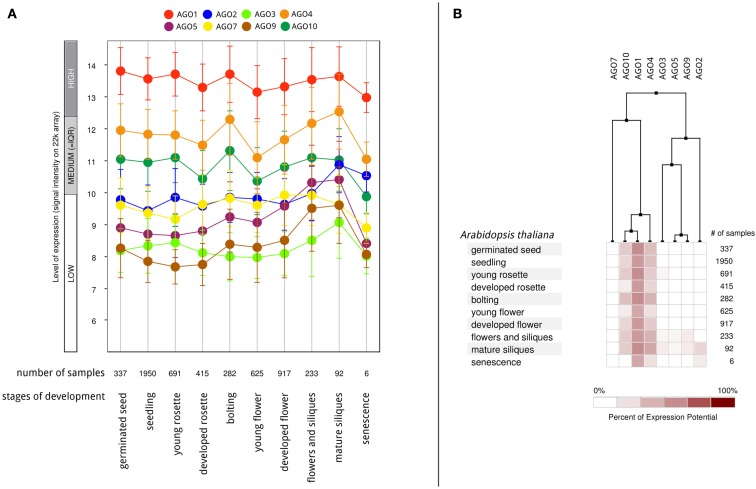
**Differential AGO expression in different stages of *A. thaliana* development based on Genevestigator microarray data**. **(A)** The level of AGO expression (log_2_-scale) at different stages of development and **(B)** hierarchical clustering on the developmental expression profiles.

**Figure 7:**
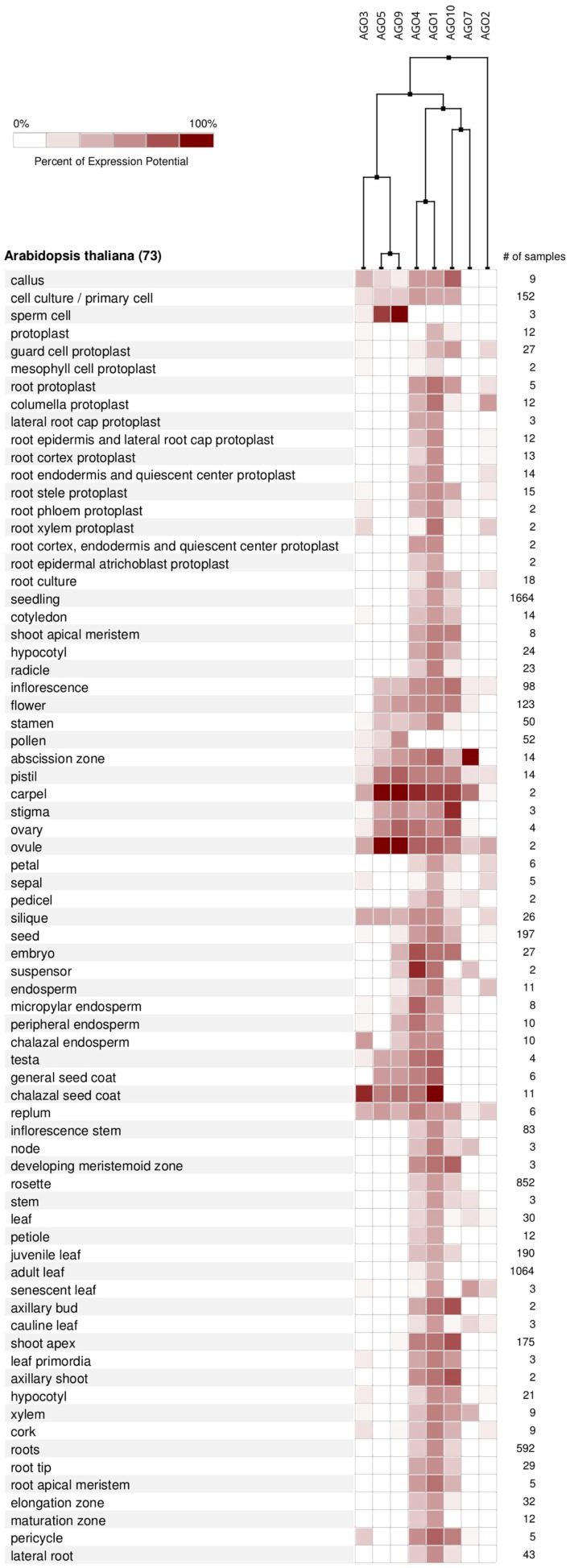
**AGO expression levels in different anatomical parts of *A. thaliana* based on microarray data from Genevestigator and associated hierarchical clustering**.

According to the available gene expression data in Genevestigator (Hruz et al., 2008), *Arabidopsis* AGOs are expressed in all organs and during all stages of plant development from the seedling to flowering stages and senescence. Among all AGO proteins, AGO1 is expressed at the highest levels and most ubiquitously, followed by AGO4 and AGO10 (Figure 6A). Overall expression levels of AGO2, AGO5, AGO7, AGO3, and AGO9 are comparatively low. Despite showing active expression, there is clear evidence of differential expression of particular AGO transcripts associated with different developmental stages (greater than twofold differences) as well as tissues and organs. The AGOs considered in this study, AGO1, 2, and 5, segregate into different groups when clustered according to expression level in different developmental stages (Figure 6B). Likewise, their expression level differs noticeably across different *Arabidopsis* tissues and organs (Figure 7). Interestingly, the expression of AGO1 and AGO5 appears quite different despite their assignment to the same phylogenetic clade based on their protein sequence. By comparison, the expression of AGO2 appears most different compared to both AGO1 and AGO5.

For AGO expression to be relevant for sorting, as a necessary condition AGO proteins ought to display differential expression. Thus, as they do indeed demonstrate differential expression, the available expression data leave open the possibility that sorting and the AGO-specific biological action may be mediated, or possibly fine-tuned by the levels of AGO transcripts, and thus, AGO proteins. However, further experimental evidence in conjunction with actual miRNA action is required to further clarify the relevance of differential AGO expression.

## Discussion

In all species with an active miRNA machinery, the processing of miRNAs and the exertion of their function requires their interaction of with AGO-based RISCs (Vaucheret, 2008; Capitao et al., 2011). As there are typically several different AGO proteins encoded in the species’ genomes the question arises whether the processing of miRNAs by the different AGOs has any functional significance, and if it does, how the sorting of miRNAs to their respective AGOs is encoded. In this study, we exploited large-scale NGS co-IP data to revisit the issue of AGO sorting in *A. thaliana*. Most importantly, we found evidence for the significance of sequence positions other than the 5′-position alone for the sorting decision.

In the following, we wish to discuss our findings in the context of reported experimental findings, point out limitations, and open questions.

### Dataset size is limiting

As a note of caution, we first address the issue of dataset size. The analyzed AGO co-IP dataset included 148 annotated mature miRNA sequences. For 126 miRNAs, specific sorting to one of four AGOs (AGO1, 2, 4, and 5; though AGO4 has been omitted in many of our analyses because of its low number of specifically associated miRNAs) was observed. As we have taken statistical approaches to the identification of sorting signals, the relatively small dataset size constitutes a major difficulty for establishing significance. Furthermore, the dataset is unbalanced with AGO1-miRNAs dominating (70 miRNAs). Thus, the data situation proved limiting. Moreover, only four representatives of the 10 known *Arabidopsis* AGO proteins were covered by the experimental data. With larger experimental datasets on miRNA-AGO sorting events, revisiting the significance of individual miRNA positions will be worthwhile in the future.

A number of miRNAs (22 = 15%) contained in the dataset did not show any pronounced preference for any of the four AGO proteins for which data was available. This may suggest that either the sorting decision is irrelevant for those miRNA, or that the AGOs that they do preferentially bind to were not in the dataset.

### The AGO sorting decision is not only associated with 5′-position alone

By applying information theoretic approaches (MI, Figure 2), sequence logos (Figure 3) as well as the RF classification methodology (Table 2), our results indicate that the sorting signal is not only be confined to the 5′-sequence position, but also resides in other miRNA sequence positions and combinations thereof. Additional positions were found to be informative and characteristic sequence motifs were detectable for the different AGOs (Figure 3). Here, uridine residues, already reported to be informative at the first sequence position, were also found to be the characteristic base type for AGO2 and AGO5 as well, but at different positions. The increased uridine frequency may not be a coincidence as uridine has been shown to exhibit an increased propensity to interact with proteins (Jeong et al., 2003).

The strength of the RF classification methodology lies in the potential to identify higher-order sorting signals beyond the univariate information, where positions are examined individually and any interactions between them are ignored. For example, our data set contained six AGO1 sequences with a 5′-cytidine instead of uridine that is otherwise typical for AGO1 (Table 1). However, another set of six sequences also starting with a cytidine are sorted to AGO5. Obviously, the decision based on position 1 alone remains inconclusive in this case. A closer inspection revealed that if those 5′-cytidine sequences harbor a guanine or uridine at position 9, they are sorted to AGO1. Otherwise, if adenine or cytidine is found at position 9, they are sorted to AGO5. Thus, in the example, the sorting decision is a conditional combination of two sequence positions. Such nested signals cannot be described by MI or sequence logos (Figure 3), but are best captured by decision trees as applied here in the form of RF.

Evidently, the finding that sequence positions other the 5′-position alone are informative for the AGO sorting calls for experimental verification. For example, it would be worthwhile to experimentally test the importance of position 9 as an additional sorting signal in 5′-cytidine sequences as discussed above.

Despite reports showing the opposite (see below), features associated to the secondary structure of miRNAs; i.e., the base-pairing across the miRNA:miRNA* duplex, were not found to be informative in the approach pursued here (Table 2).

It is conceivable that covalent modifications such as methylation of RNA bases expand the code for AGO sorting. However, apart from the observed methylation of the 3′-end of plant miRNAs to prevent rapid degradation (Fang and Spector, 2007), no such modifications have been reported yet.

### Impact of miRNA sequence length

We found no significant contribution of miRNA sequence length to the AGO sorting predictions as also the respective lengths distributions were found to be very similar (Figure 1). Experiments for AGO4, AGO6, and AGO9 also demonstrated independence of sequence length (Havecker et al., 2010). Rather, as reported in (Vaucheret, 2009) for 21-nt and 22-nt isoforms of miR168 miRNA, changes in miRNA length were shown to influence the downstream efficiency of the RISC. Single nucleotide extensions, such as the 5′-extension of the mature miRNA by uridine, introduce additional changes in miRNA length (Ebhardt et al., 2010). *In silico* analyses for miR156h and miR775 imply that such extensions are able to redirect from AGO1 toward AGO5. It is unclear whether this observation is caused by changes at the 5′-end or 3′-end, as the whole miRNA sequence is shifted within the AGO protein/RISC. Similarly, 3′-additions were shown to affect binding affinities of human miRNAs to AGO2 and AGO3 (Burroughs et al., 2010). A final conclusion of the relevance of miRNA length on the AGO sorting will require larger datasets including more AGO types than considered here.

### AGO loading – single stranded miRNA or miRNA:miRNA* duplex?

The nature of the actual RNA molecule – AGO protein recognition and binding process, and more specifically, the question whether the AGO protein binds a single or double-stranded RNA molecule is crucial for the understanding of the AGO sorting process and the search for sorting signals. There are two hypotheses as to when the separation of miRNA from the associated star strand is occurring. Here, we are referring to them as “loading first” and “unwinding first.”

The “loading first” would proceed by first loading the whole miRNA:miRNA* duplex into the RISC. In a second step, the selection and separation of the actual miRNA and star strand is performed (Iki et al., 2010; Kawamata et al., 2011; Manavella et al., 2012). A number of experimental findings are consistent with this mode of AGO loading as properties associated specifically with the duplex and not the single miRNA strand have been found to be responsible for the AGO sorting and function. In *Drosophila melanogaster*, the sorting of double-stranded small RNAs to either AGO1, mediating the miRNA pathway, or AGO2, routing small RNAs into the RNAi pathway, was observed to depend on the presence (in the case of AGO1) or absence (AGO2), respectively, of a central mismatch in the duplex (Forstemann et al., 2007; Tomari et al., 2007; Kim, 2008). In *Caenorhabditis elegans*, introducing mismatches into the duplex was observed to lead to a redirection of small RNAs from the RNAi- to the miRNA pathway (Steiner et al., 2007). In *A. thaliana*, similar effects have been detected. For example, the miR165 and miR166 families were surmised to be bound by AGO10 as opposed to AGO1 because of the higher number of unpaired bases than can be tolerated by AGO1 (Zhu et al., 2011). Similarly, asymmetric bulges in the duplex structure have been shown to trigger the production of secondary siRNA in AGO1 instead of target cleavage (Manavella et al., 2012). Furthermore, it has been proposed that the duplex stabilizes the established RISC complex (Kawamata et al., 2011) and AGO1 extracted from tobacco protoplasts were shown to bind RNA-duplexes with subsequent unwinding and removal of the miRNA* molecule (Iki et al., 2010). Also mechanistically, it was possible to associate the necessary unwinding of the duplex with the N-terminal AGO domain (Kwak and Tomari, 2012).

In the second possible AGO loading mode, “unwinding first,” duplex unwinding and dissociation happens first. Subsequently, single stranded small RNAs, such as different subclasses of siRNAs, are recognized by AGO (Chapman and Carrington, 2007; Lee et al., 2012). In some cases, unpaired miRNA star strands are not degraded, but consumed by another AGO complexes (Devers et al., 2011; Zhang et al., 2011). In this mode, duplex-derived features should not be informative for the sorting and the sorting signal should lie primarily in the RNA sequence instead. Another argument in favor of binding single stranded RNA molecules comes from structural considerations. Adopting an A-RNA helical structure, the miRNA:miRNA* duplex would complete nearly two full turns. Therefore, the unwinding and dissociation of the duplex seems sterically challenging with within the protein complex.

### The relevance of structural patterns of the miRNA:miRNA* duplex

Efficient AGO sorting may not only rely on the identity of nucleotides at a particular position along the sequences of small RNAs. Assuming “binding first,” the pattern of base-pairing in the miRNA:miRNA* duplex may serve as a sorting signal as well. Unpaired or even bulged out nucleotides may be structurally less constrained, and therefore are free to engage in specific interactions with an AGO protein. For example, the 5′-nucleotide of miRNAs has been described to rotate out of the duplex and into the MID binding pocket establishing base- and AGO-specific spatial interactions (Wang et al., 2008; Frank et al., 2012).

All Ath-miRNAs contained in miRBase are derived from miRNA:miRNA* duplexes with high degree of canonical base-pairing allowing the formation of robust helical structures. Interestingly, positions 1, 10–13, and 21, where mismatches are tolerated most frequently (Figure 4), correspond to positions of increased MI values (Figure 2) implying that at those positions more structural flexibility is tolerated or even required to meet potential AGO-specific sequence requirements.

However, multivariate feature selection by RF did not reveal any significant impact of the base-pairing pattern on the sorting decision in the dataset used here. This result suggests that duplex-related structural features brought about by base-pairing may not be relevant in the “loading first” scenario and that the “unwinding first” mode cannot be ruled out based on the argument of required structural features associated with the duplex molecule. Furthermore, it has to be borne in mind that our dataset comprised only three (as used in the RF predictions) of the 10 *Arabidopsis* AGOs and both recognition modes (single or double-stranded RNA) may coexist depending on the AGO and small RNA molecule. Base-pairing patterns may still turn out to be relevant once comparative information for more AGO types becomes available.

### AGO recruiting or stabilization by additional protein factors – motifs in flanking sequence regions?

miRNAs are shown to contain several cis-regulatory elements even within the precursor molecule (Piriyapongsa et al., 2011) and interactions with proteins occur during various phases of miRNA maturation such as the processing by the protein DCL1, methylation by HEN1 (HUA ENHANCER1), and the export from the nucleus (Lobbes et al., 2006; Chapman and Carrington, 2007; Axtell et al., 2011; Mateos et al., 2011). Also, viral RNA suppressor proteins have been shown to interfere with miRNA processing (Chapman et al., 2004; Schott et al., 2012). It is to be assumed that throughout their lifetime, small RNAs are accompanied and protected by several proteins.

In *A. thaliana*, the protein DRB1 (HYL1) is shown to assist strand selection and AGO1 loading (Eamens et al., 2009) and in Drosophila, R2D2 is important for the redirection of endo-siRNAs with a central mismatch to the AGO2-mediated RNAi pathway (Okamura et al., 2011). Such additional proteins could potentially recognize up- and downstream sequence and thus guide AGO recruitment or contribute to the stabilization of the complex. However, our scans for such motifs using established motif finding algorithms (Meme and Amadeus) did not turn up any candidate motifs indicative of any additional AGO-specific factors.

Notwithstanding these observations, it is very likely that additional, and as of yet undetected protein interactions may occur. For example, miR159, miR165, miR166, and miR168 are usually incorporated into AGO1-based RISCs, but associate with other AGOs in AGO1-deficient *Arabidopsis* mutants, where this redirection is supposed to be mediated by stabilizing proteins (Vaucheret, 2009; Zhu et al., 2011).

### Differential spatial or temporal expression of AGOs and miRNAs might assist in AGO sorting

miRNAs are under the control of various, but highly specific promoters elements generating clear patterns of differential expression in developmental stages as well as tissue localization (Valoczi et al., 2006; Figures 6 and 7). These observations suggest that differential expression may influence the AGO sorting.

From the expression-based dendrogram shown in Figures 6 and 7, we conclude that AGO1 and AGO4 are essential for most miRNA and siRNA pathways as they are both consistently expressed at high levels. In addition, AGO10 and AGO7 belong to this cluster. Both have been demonstrated to selectively withdraw small RNAs from AGO1 pools and thus are likely coupled to the expression of AGO1 (Montgomery et al., 2008; Mallory et al., 2009; Zhu et al., 2011). Another cluster is formed by AGOs of probably minor importance as judged by their expression level, which may mediate tissue and time specific regulatory functions. Other observations, such as AGO expression being influenced by small RNAs via of negative feedback loops (Mallory and Vaucheret, 2010), further highlight the relevance of AGO expression for small RNA regulation and function. Beyond expression level, the activity and function of AGO proteins may also be altered by covalent modifications such as phosphorylation or other post-translational modifications, which remains to be investigated.

## Conclusion

The sorting to different AGO proteins appears to influence the fate and function of miRNAs. Based on a set of miRNAs with experimentally verified AGO sorting preferences in *A. thaliana*, we found that in addition to the 5′-position of miRNAs, the remainder of the miRNA sequence also carries information with regard to the sorting decision. Thus, the apparent conflict of a greater number of different AGOs than can be encoded by the four different bases at the 5′-position may find its solution in additional informative positions across the entire miRNA sequence. Particular relevance may be associated with positions 2, 6, 9, and 13 as identified here via the applied MI and RF variable importance metric. Furthermore, uracil bases at defined positions appear to be important for the sorting to AGO2 and AGO5, in particular. By contrast, we did not find any evidence of the presence of additional motifs in the flanking sequence of miRNAs, nor any indication for a length- or base pair binding-pattern-based sorting mechanism. In addition to miRNA sequence influencing the sorting, the temporal and spatial expression patterns of the different AGO proteins likely contribute to the fine-tuning of miRNA function. The results reported in this study await further validation once larger datasets covering all 10 known AGO proteins in *Arabidopsis* as well as data for different species will become available.

## Conflict of Interest Statement

The authors declare that the research was conducted in the absence of any commercial or financial relationships that could be construed as a potential conflict of interest.
